# Systematic review of the scientific evidence of the pulmonary carcinogenicity of talc

**DOI:** 10.3389/fpubh.2022.989111

**Published:** 2022-10-11

**Authors:** Heather N. Lynch, Daniel J. Lauer, William J. Thompson, Olivia Leleck, Rachel D. Freid, Justin Collins, Kathleen Chen, A. Michael Ierardi, Ania M. Urban, Michael A. Cappello, Paolo Boffetta, Kenneth A. Mundt

**Affiliations:** ^1^ChemRisk (Stantec), Boston, MA, United States; ^2^Population Sciences, Stony Brook Cancer Center, Stony Brook, NY, United States; ^3^Department of Medical and Surgical Sciences, University of Bologna, Bologna, Italy

**Keywords:** systematic review, talc, hazard assessment, carcinogenicity, risk assessment, lung cancer, mesothelioma

## Abstract

We conducted a systematic review to assess the potential pulmonary carcinogenicity of inhaled talc in humans. Our systematic review methods adhere to Preferred Reporting Items for Systematic Reviews and Meta-Analyses (PRISMA) guidelines and incorporated aspects from the US Institute of Medicine (IOM) and several United States (US) Environmental Protection Agency (EPA) frameworks for systematic reviews. A comprehensive literature search was conducted. Detailed data abstraction and study quality evaluation, adapting the US Toxic Substances Control Act (TSCA) framework, were central to our analysis. The literature search and selection process identified 23 primary studies that assessed exposure to talc and pulmonary cancer risks in humans (*n* = 19) and animals (*n* = 3). Integrating all streams of evidence according to the IOM framework yielded classifications of suggestive evidence of no association between inhaled talc and lung cancer and pleural mesothelioma at human-relevant exposure levels.

## Introduction

Talc is a hydrous magnesium sheet silicate (Mg3Si4O10(OH)2) with particles that are plate-like in structure. Mined mineral talcs may contain various amounts and forms of accessory minerals. It has been reported that some cosmetic talcs and finished talcum powders may contain trace levels of asbestiform minerals despite the lack of evidence of reliably detectable asbestos at the major sources (1). However, there have been challenges with accurately identifying and quantifying asbestiform minerals in talc (2, 3). As a result, the validity and relevance of these findings remains unclear; however, the epidemiological studies reflect potential risks associated with exposures to talcs including whatever accessory minerals and contaminants that might be present.

In its most recent review of talc, the International Agency for Research on Cancer (IARC) concluded “[t]here is *inadequate evidence* in humans for the carcinogenicity of inhaled talc” (4). They also concluded “[t]here is limited evidence in experimental animals for the carcinogenicity of talc not containing asbestos or asbestiform fibers.” This review is now 12 years old, and several studies and reviews on talc exposure and pulmonary cancer risk have been published since the 2010 IARC Monograph (1, 5, 6). The objective of this paper was to apply systematic review methods to critically evaluate and synthesize the scientific evidence addressing the possible relationship(s) between exposure to talc occupationally and from exposure to talc-containing products (primarily talcum powders and cosmetics) and pulmonary cancers, specifically lung cancer and pleural mesothelioma, integrating epidemiology, toxicology, and studies informing potential underlying modes of action.

## Materials and methods

This systematic review was conducted in accordance with the Preferred Reporting Items for Systematic Reviews and Meta-Analyses (PRISMA) checklist, using a “hybrid” systematic review framework that incorporates aspects from several recognized systems. We relied most heavily on the U.S. Environmental Protection Agency's (EPA) protocol for systematic reviews conducted under the Toxic Substances Control Act (TSCA) and the Draft Handbook for the Integrated Risk Information System (IRIS) (7). Hazard conclusions were determined using the U.S. Institute of Medicine (IOM) framework (8). An overview of our methods is provided below, and an example of their application can be seen in a recent review on ethylene oxide (9). Our evaluation of reproductive tract cancers, following the same methods as the current review, to be presented in a companion manuscript (10). Additional details of our methodology are provided in the Protocol in the Supplementary material, allowing verification and replication of our review.

We developed *a priori* inclusion and exclusion criteria to identify the most relevant articles for full review consistent with systematic review principles. In brief, selected literature pertained to talc exposure *via* inhalation and addressed potential associations with lung cancers or pleural mesothelioma. We included epidemiological studies, experimental animal studies in mammalian species, and mechanistic studies *in vivo* or in mammalian or bacterial cell lines. We performed literature searches using PubMed and Web of Science and used existing agency reviews as a basis for cross-referencing critical studies. The preliminary search string was as follows: (talc OR “talcum powder”) AND (“cancer” OR “carcinogen” OR “mesothelioma”). Additional searches were run using filters for animal/toxicology studies, and for mechanistic/mode of action (MOA) studies, using search terms including, but not limited to the following: micronuclei, sister chromatid exchange, chromosome aberrations, DNA adduct, DNA methylation, inflammation, mechanism, and MOA.

Experimental animal and mechanistic studies were selected based on their overall relevance to chronic health effects (primarily tumor formation and cancers) and adherence to the Population, Exposure, Comparator, and Outcome (PECO) criteria. Epidemiological studies were selected to include groups exposed to talc, including talc miners and millers (the groups historically most highly exposed) as well as groups potentially exposed to cosmetic talcum powders and other products containing talc. Each study was reviewed for relevance, and if the full text met inclusion criteria, study information was extracted into tables and the study was evaluated for reporting and methodological quality.

**Figure 1:**
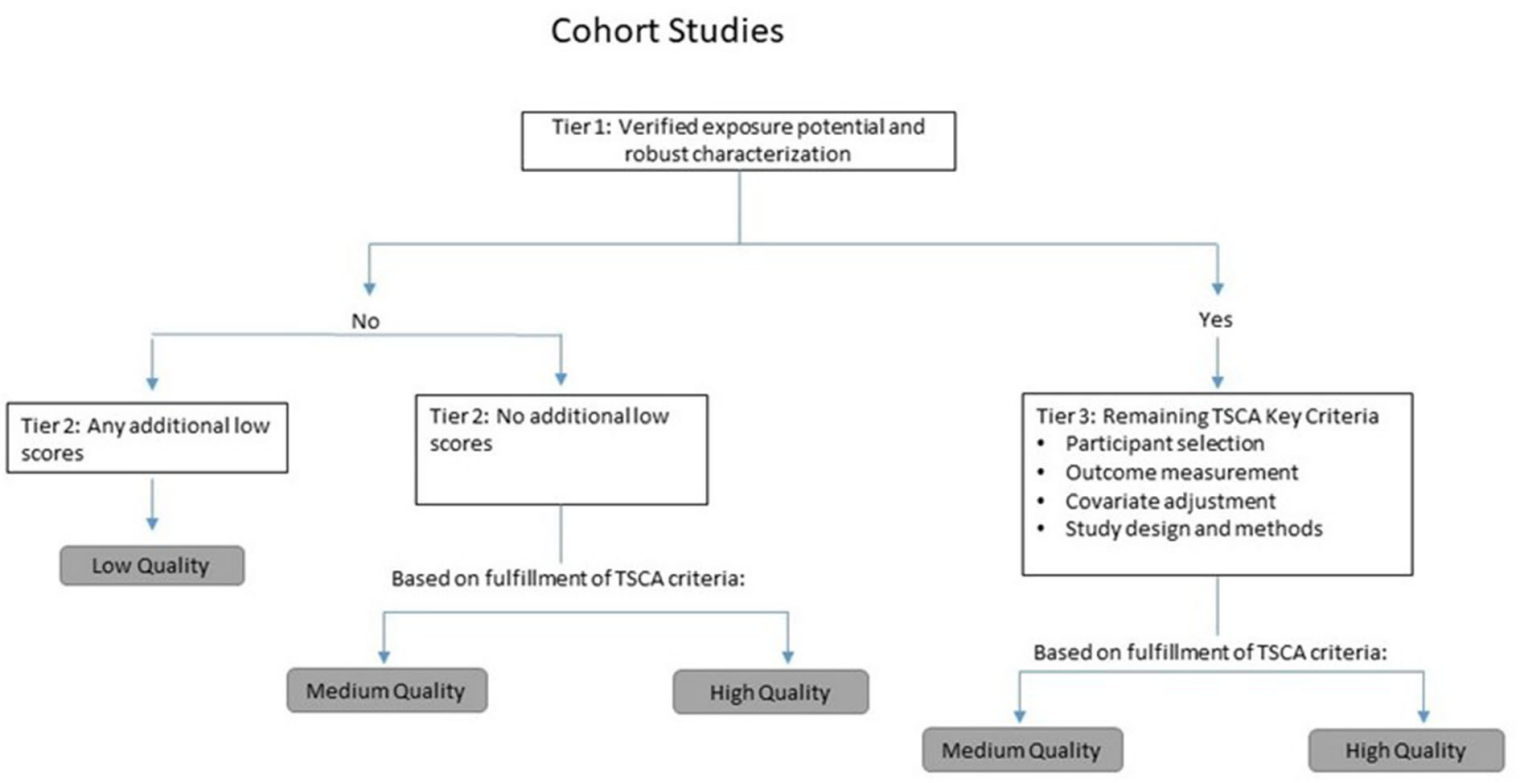
Tiering approach used in evaluating cohort studies.

We followed a modified version of the study quality framework used by U.S. EPA TSCA risk evaluations (11, 12). Specifically, this framework involves reviewing and evaluating studies according to specific quality domains (e.g., outcome assessment and exposure characterization), each of which includes two to seven individual metrics assessing specific study features. All studies were screened and evaluated by two independent reviewers, and any discrepancies discussed and resolved. We employed qualitative and hierarchical or tiered approaches to arrive at the overall study quality score, tailored to each study type and outcome. The tiering system allows for preferentially weighting specific quality domains (e.g., exposure characterization) first, followed by more secondary determinants of study quality. A flow chart for the overall tiering approach used for cohort studies is provided as an example in Figure 1.

For animal toxicology and selected mechanistic studies, we followed the TSCA study quality evaluation framework (12, 13) and assigned relative numerical ranks to each of the outcomes (1, 2, and 3 corresponding to high, medium, and low) for each metric, then averaged the metric scores to arrive at an overall relative rating of high, medium, or low quality. Mechanistic studies were evaluated according to Klimisch scoring (14).

Evidence was synthesized across all epidemiological studies and then integrated with animal study findings and mechanistic considerations to reach conclusions for human pulmonary cancers. Integration of evidence included consideration of consistency, coherence and the presence of exposure-response relationships. Overall conclusions were derived for each cancer: sufficient evidence of a causal relationship; sufficient evidence, suggestive evidence, or inadequate/insufficient evidence of an association; or suggestive evidence of no association. The nomenclature of these classifications is simplified but follows the rationale of the corresponding U.S. IOM classifications for causation (8) (Table 1).

**Table 1:** Categorizations for evaluating strength of evidence^a^ (8).

**Classification**	**Description**
Sufficient evidence of a causal relationship	Evidence is sufficient to conclude that a causal relationship exists between the exposure to a specific agent and a health outcome in humans. The evidence fulfills the criteria for sufficient evidence of an association (below) and satisfies several of the criteria used to assess causality: strength of association, dose-response relationship, consistency of association, temporal relationship, specificity of association, and biological plausibility.
Sufficient evidence of an association	Evidence is sufficient to conclude that there is a positive association. That is, a positive association has been observed between an exposure to a specific agent and a health outcome in human studies in which chance, bias, and confounding could be ruled out with reasonable confidence.
Limited/suggestive evidence of an association	Evidence is suggestive of an association between exposure to a specific agent and a health outcome in humans, but is limited because chance, bias, and confounding could not be ruled out with confidence.
Inadequate/insufficient evidence to determine whether an association does or does not exist	The available studies are of insufficient quality, consistency, or statistical power to permit a conclusion regarding the presence or absence of an association between an exposure to a specific agent and a health outcome in humans.
Limited/suggestive evidence of no Association	There are several adequate studies covering the full range of levels of exposure that humans are known to encounter, that are mutually consistent in not showing a positive association between exposure to a specific agent and a health outcome at any level of exposure. A conclusion of no association is inevitably limited to the conditions, levels of exposure, and length of observation covered by the available studies. In addition, the possibility of a very small elevation in risk at the levels of exposure studied can never be excluded.

Source: IOM (8).

^*a*^IOM has since updated the classification language, but the same general underlying considerations for reaching each conclusion. The previous classification categories were retained as we believed the previous categories were more easily interpreted than the updated categories.

## Results

### Literature search and selection

**Figure 2:**
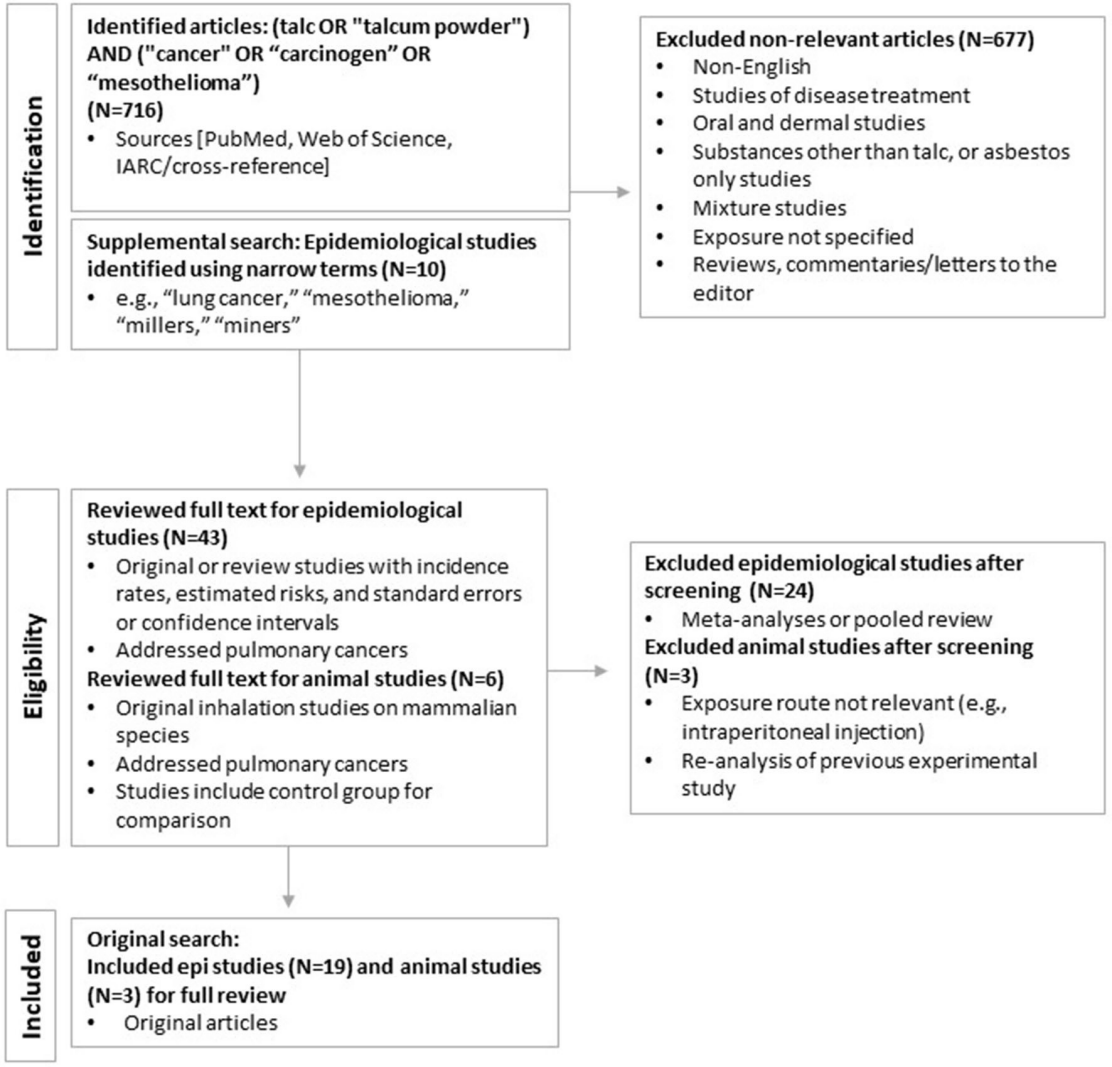
Literature search and selection process.

The primary literature search for talc exposure and all cancers was performed in PubMed (in April; updated September 2021) and yielded a total of 716 publications. After eliminating duplicates or studies that were subsequently updated, and applying the inclusion and exclusion criteria determined *a priori*, 43 epidemiological and six animal studies remained for detailed review. An additional 10 epidemiological studies were identified through supplemental searches using narrower terms (e.g., “lung cancer,” “mesothelioma,” “millers,” “miners”) and considering the tertiary literature (reviews, gray literature). Additional searches in Web of Science identified no additional publications. Of these, 19 epidemiology studies and three animal studies were determined to address pulmonary cancers and included in this systematic review. The results of the literature search and study selection process are summarized in Figure 2.

### Pharmacokinetics of talc in the respiratory system

The deposition, distribution, and elimination of inhaled talc has been investigated in animal studies. Generally, aerosolized talc has an alveolar biological half-life of about 7–10 days in animals. In one study, Syrian golden hamsters were administered a single, 2-h, nose-only exposure to commercial baby powder (MMAD of 6.4–6.9 μm) (15, 16). Between 6% and 8% of the inhaled dose was deposited in alveoli. By 132 days after exposure, there were no statistically significant differences in talc burden in the lungs of exposed vs. unexposed hamsters, indicating clearance of the particles.

### Experimental animal studies

Our search identified six studies assessing talc exposure and tumor formation in animal models. After excluding studies based on exposure route, non-pulmonary outcome, and administration of talc as part of a mixture, three studies remained for study quality evaluation (17–19). Study details can be found in Supplementary Table 1. Based on the quality evaluation methods (see Materials and methods), the three studies assessing talc toxicology in animals were rated as high quality. A brief overview of the quality evaluation results is presented below, followed by a summary of study findings. Full quality evaluation results are presented in Supplementary Table 2.

#### Quality evaluation results

Overall, the animal studies all employed designs that properly incorporated a control group for comparison, including randomly allocating test animals into experimental groups to reduce potential bias. All three studies provided explicit descriptions of talc aerosolization with exposure consistently administered across both control and experimental groups (except for some fluctuations in the NTP study). Outcome assessment for these studies typically encompassed a full histological examination or autopsy; clinical and body weight monitoring was also conducted in two of the studies (15, 18, 19). Although these studies overall were considered to be of high quality, there were some important deficiencies that limited their utility, which are discussed below.

#### Summary of study findings

Wehner et al. (15, 18) exposed groups of 100 Syrian golden hamsters to whole-body inhalation of Johnson's Baby Powder at 8 mg/m^3^ for 3, 30, or 150 min/day, for 5 days/week for 30 days, or for 30 or 150 min/day, 5 days/week, for up to 300 days. Two groups of 50 hamsters also were exposed to laboratory air as a control for 30 or 300 days. Calculated cumulative talc exposures were 12, 120, and 600 mg-h/m^3^ for 30 days or 1,200 and 6,000 mg-h/m^3^ for the 300-day groups. Exposure to talc aerosol produced no statistically significant differences in body weight; survival; or the type, incidence, or degree of histopathological change, relative to unexposed controls. There was one lung carcinoma found in the group exposed to talc for 3 min/day for 30 days, and one lung carcinoma found in the 300-day control group. However, the authors noted that these tumors were metastatic and that there were no primary neoplasms found in the respiratory system.

Wagner et al. (17, 20) exposed Westar rats to Italian 00000 grade talc, superfine chrysotile asbestos, or laboratory air (controls), *via* whole-body inhalation. Talc concentrations of 10.8 mg/m^3^ were administered for 7.5 h per day, 5 days per week; 48 animals from each group were exposed for 3 months, 24 animals for 6 months, and 24 animals for 12 months with cumulative doses of approximately 4,100, 8,200, and 16,400 mg/m^3^-h for the 3-month, 6-month, and 12-month exposure durations, respectively. Two adenomas (not considered cancerous) were observed in talc-exposed animals, 13 lung tumors were found in chrysotile-exposed animals, and one adenoma occurred in the air-exposed control group. No statistical tests were reported in either publication. In both the talc and chrysotile exposure groups, fibrosis was observed to a similar extent, with minimal to no fibrosis observed in the air-exposed control group.

The National Toxicology Program (19) exposed F344/N rats and B6C3F1 mice to air or micronized Pfizer MP 10–52 talc via whole-body inhalation. Animals were exposed to 0, 6, or 18 mg/m^3^ talc for 6 h/day for 5 days/week until death or until the mortality of any exposure group reached 80% (approximately 2 years). There was no evidence of carcinogenic activity of talc in male or female B6C3F1 mice. Generally, lung talc burdens of mice exposed to 18 mg/m^3^ were disproportionately greater than those of mice exposed to 6 mg/m^3^, suggesting clearance of talc from the lung was impaired in mice exposed to 18 mg/m^3^. In male F344/N rats, there was an increased incidence of benign or malignant pheochromocytomas of the adrenal gland, commonly observed in rats (21). In female rats, there was an increased incidence of alveolar/bronchiolar adenomas and carcinomas of the lung and pheochromocytomas of the adrenal gland. There was a single occurrence of malignant mesothelioma in a high-dose male rat; however, aging F344 rats have been reported to develop spontaneous mesotheliomas (22). In both rats and mice, 2-year inhalation exposure to talc was associated with chronic active inflammation and accumulation of macrophages in the lung (19). The authors reported issues with the consistency of administration of talc aerosol over some weeks in the middle of the study, likely resulting in increased talc exposure. Additionally, the NTP (19) study involved the use of micronized talc, which has a smaller aerodynamic diameter than mined and milled talcs and talc used in talcum powder products. While this does not affect the quality of the study, as discussed below, micronized talc is not used in cosmetic talcum powder products and may limit the study's generalizability for assessing risk associated with the use of cosmetic talcum powder.

### Summary and conclusions for animal evidence

Across the animal studies assessing chronic talc toxicity, results largely demonstrate a lack of talc carcinogenicity. Although two of the studies reported no increase in tumor formation among talc-exposed animals (15, 17, 18, 20), NTP (19) reported a higher incidence of lung tumors in female rats in the highest exposure group, relative to controls. However, the lung tumors occurred only in female rats exposed at a dose also inducing significant chronic lung toxicity and high lung talc burden, such that the maximum tolerable dose may have been exceeded (21). Particle overload is common when high doses of poorly insoluble particles are administered – particle clearance mechanisms of the lung are overwhelmed and carcinogenic processes are initiated (23, 24): see “Mechanistic evidence and Mode of Action.” There was no evidence of carcinogenicity in mice exposed to talc. Overall, in available animal studies, there is indeterminate evidence that talc is associated with lung tumors in rodents based on negative findings in several high-quality studies and species, but positive results in a single species and sex (female rats) exposed to high doses of micronized talc that caused particle overload conditions.

### Mechanistic and mode of action evidence

#### Genotoxicity

Talc was negative for mutagenicity and other forms of genotoxicity in all available assays, described in brief below.

In an OECD Guideline 473 *in vitro* mammalian chromosome aberration test (rated as a Klimisch score of two), Endo-Capron et al. (25) reported that talc did not cause significantly increased frequency of sister-chromatid exchange or increased DNA repair synthesis in rat pleural mesothelial cells, relative to positive or negative controls (16).

Talc was negative in a reverse mutation assay *Salmonella typhimurium* strains TA1530 and G46. Talc was also negative in a companion host-mediated mutagenicity assay using male ICR mice injected with *Salmonella typhimurium* and *Saccharomyces cerevisiae*, with and without metabolic activation (16).

In an *in vivo* OECD Guideline 478 Dominant lethal test, male Sprague-Dawley rats were exposed *via* gavage to a single dose or one dose/day for 5 days of 300, 3,000, or 5,000 mg/kg talc. No chromosomal aberrations in the bone marrow or dominant lethal mutations at any dose were observed (16).

#### Inflammation

As with other particles, one postulated carcinogenic MOA for talc is chronic inflammation resulting from the overwhelming of particle clearance mechanisms (i.e., phagocytosis), long-term tissue irritation and release of inflammatory chemokines and cytokines, and reactive oxygen species (ROS) formation. For pulmonary cancers, it is therefore plausible that talc could cause irritation and a cascade of inflammatory mechanisms.

Beck et al. (26) sonicated respirable granite and talc dust from a talc mine in Vermont (0.8 μm, MMAD of 7.5 μm) in saline and intratracheally instilled it into the lungs of Syrian Gold Hamsters at doses of 0.15, 0.75, or 3.75 mg/100 g (single exposure). Bronchioalveolar lavage (BAL) fluid was collected and assessed at 1 day post exposure (all doses) or 1, 4, 7, and 14 days after talc administration (3.75 mg/100 g). One day after dosing, macrophage numbers were not significantly altered, but there were increases in polymorphonuclear leukocytes (PMNs), lactate dehydrogenase, and peroxidase, indicating cellular injury. Albumin, a marker of pulmonary edema, also was increased. In the time-course evaluation, most findings returned to control levels within 14 days. However, macrophage numbers decreased in days 4–14, and remained decreased, indicative of a chronic effect. Note that this route of administration (intratracheal instillation) produces different lung distribution patterns compared to inhalation, which limits its relevance to inhalation exposures to talc in humans.

Pickrell et al. (27) exposed F344/Crl rats and B6C3F1 mice to unspecified talc *via* whole-body inhalation and compared tumor development in these animals relative to non-exposed rats and mice as controls. Talc was administered to both species for 6 h per day, 5 days per week, for a total of 20 exposure days at talc concentrations of 0, 2, 6, or 18 mg/m^3^. Histologic evaluation of lung tissue revealed no exposure-related lesions except for a modest, diffuse increase in free macrophages within alveolar spaces of both rats and mice exposed to the highest concentration of talc. In mice, intra-alveolar macrophages were focally aggregated. The normalized lung talc burdens for both mice and rats were lower at the lowest exposure level than at the two higher exposure levels; however, the difference was statistically significant only for the rats.

Similar findings were reported in rats exposed to non-asbestiform talcum powder for 6 h per day for 4 weeks *via* whole-body inhalation (28). The authors observed increases in macrophage infiltration at 50 and 100 mg/m^3^ talc, but not at the lowest concentration of 5 mg/m^3^. Markers of oxidative stress (superoxide dismutase 2) were increased at 100 mg/m^3^.

### Conclusions for mechanistic and MOA evidence

The pharmacokinetic data on the fate of inhaled talc indicate rapid clearance from the lung and body after single doses and no translocation of talc to other organs after single or repeated exposures. The evidence for possible carcinogenic mechanisms of talc is limited; however, a genotoxicity MOA confidently can be ruled out. A few studies provide evidence of some key events in the proposed inflammatory MOA; however, data are limited to non-human relevant exposure pathways and/or cell-based assays.

Markers of inflammation also have been observed in the lungs of rats after talc exposure, but one of the only available mechanistic studies utilized a route of exposure (i.e., intratracheal instillation) that is not comparable to inhalation exposures. Further, some aspects of the physiology and function of the respiratory system of rodents (e.g., a delicate balance of pulmonary surfactants, as well as smaller and fewer macrophages relative to humans) make them highly susceptible to high doses of solid particles relative to humans (29). Overall, the mechanistic evidence is insufficient to support an MOA whereby talc induces pulmonary carcinogenesis.

### Epidemiological studies

Nineteen epidemiological studies (17 cohort study publications and two nested case-control studies) satisfied inclusion and exclusion criteria and were selected for full review to assess the possible relationship between occupational talc exposure and pulmonary cancers (specifically lung cancer and mesothelioma). Several of these publications were updates of reports based on the same cohorts. Two nested case-control studies were conducted in tandem with occupational mortality analyses (30, 31).

We also identified several meta-analyses/pooled studies, two large cancer registry-based linkage studies examining cancer risks by occupational group but lacking specific information on individual exposures (32, 33), and several talc pleurodesis studies (34–37). None of these studies met our inclusion criteria and therefore did not undergo full study quality review. No epidemiological studies were identified that assessed consumer use of talc and talcum powder products. Three case series (38–40) drawn from medico-legal consultation practices were identified, but these did not meet the inclusion criteria for epidemiological studies (e.g., because they lack referent or control groups) and thus were not selected for further review.

Therefore, the body of literature eligible for full review consisted of studies evaluating workers occupationally exposed to high levels of talc during mining and milling operations. The 17 occupational cohort study reports evaluated for study quality encompassed talc miners and millers in New York State (41–47), Italy (1, 48–51), Norway (52, 53), France and Austria (30) and Vermont (54–56). Details regarding the cohorts and study methods can be found in Supplementary Tables 3, 4. Briefly, the cohorts ranged in size from about 400 (53) to over 1,700 (1) miners and millers that were followed for mortality for up to seven decades. For each cohort, excluding the Austrian and French cohorts, mortality was updated at least once.

The Italian, Norwegian, French, and Austrian talc mines all produced talcs described as non-asbestiform with various accessory minerals including small amounts of chlorite and quartz (30, 50, 52). Similarly, Vermont talc miners and millers encountered talcs “free of both asbestiform mineral and significant quantities of free silica” (54, 56). Some of the mines in upstate New York were reported to produce talcs with asbestiform or non-asbestiform amphibole minerals including tremolite and anthophyllite (42, 43, 57). Historical occupational talc exposure most often was estimated using duration of employment as a surrogate. Historical air sampling records (i.e., total respirable dust) also were available at some locations. Pulmonary cancer (lung cancer and mesothelioma) mortality generally was ascertained *via* death certificates coded by certified nosologists.

Across all cohort studies, observed numbers of deaths for site-specific cancers among talc miners and millers (either combined or separately) were compared with expected numbers based on national and/or regional reference rates. When available, we focused our analysis on the most recent update that provided the most complete vital status and cause of death ascertainment.

#### Quality evaluation results

Based on the quality evaluation methods, domains and quality criteria (described above and detailed for all studies in Supplementary Tables 5, 6), most of the studies assessing pulmonary cancer risk among talc miners and millers were rated medium quality. While many studies received high ratings for individual metrics such as study participation and outcome assessment, limitations in other metrics or domains precluded some studies from receiving a high overall rating. Overall ratings of medium quality for 14 cohort studies primarily were driven by potential confounding/variable control (especially for lung cancer and cigarette smoking) and exposure assessment, including characterization of talc exposure (e.g., by job, exposure concentration level and duration). Because the case-control studies were nested within cohorts, we did not expect substantive differences in quality. We pilot tested the case-control quality evaluation for Gamble (31) and determined that its rating was equivalent to the associated cohort study (41) and thus did not separately evaluate the second case-control study.

In general, potential confounders such as age were appropriately considered in the statistical analyses, and employment records were used to extract key demographic and work history information. However, high quality ratings were rare due to inadequate information on prior employment history among the cohort members, which precluded determining the potential for prior occupational exposure to asbestos. For example, Fordyce et al. (56) reported that the death certificate for the single mesothelioma death in the cohort “explicitly mentioned exposure to asbestos”; yet no additional information was available regarding this potential exposure (56). On the other hand, Honda et al. (43) obtained relatively detailed information on prior employment histories through next of kin interviews for the two presumed (but not verifiable) mesothelioma deaths reported in that study.

#### Epidemiological findings for malignant mesothelioma

None of the 17 cohort studies identified an increased risk of malignant pleural or peritoneal mesothelioma among talc miners and millers. No mesothelioma deaths were observed in most of the cohorts, including the Austrian, French, Italian, and Norwegian talc miners and millers (1, 30, 53). In the Vermont cohort, a single mesothelioma death was reported in the latest update; however, this case was identified through a detailed review of death certificates for the cohort (which was not performed for the reference group) in a field on the death certificate different from the conventional field for underlying cause of death (56). As noted above, the talcs produced from these mines have been reported not to contain asbestiform minerals and testing has not produced reliably detectable levels of asbestos of any fiber type [e.g., regarding the Italian mines, see (1)].

Kleinfeld and coworkers published two proportionate mortality studies on miners and millers in upstate New York potentially exposed to talc with asbestiform or non-asbestiform amphibole minerals, largely in response to reports of fibrogenic pneumoconiosis (44, 45). One peritoneal mesothelioma was reported in both studies (the same individual) and analyses including this case in the category of “gastric cancers” found no excess risk overall or by specific age categories (44, 45). Methods used to derive cause of death were poorly described, and included a variety of sources including death certificates, employment records, and hospital records, resulting in a low quality rating for that domain, and low overall quality rating (44, 45).

Five additional mortality studies evaluated specific causes of death among New York talc miners and millers (41–43, 46, 47). Lamm et al. (46) reported one mesothelioma (site not specified) in an electrician hired at the plant 15 years prior to his death [possibly the same case reported in (42)]. Brown et al. (41) studied the same plant, but their NIOSH Health Hazard Evaluation Report mentioned no mesothelioma. Honda et al. (43) extended follow-up of this cohort from 1948 through 1989, reporting two deaths from pleural mesothelioma; however, the underlying causes of death on the death certificates officially were coded by the New York State nosologists as “benign neoplasm of the respiratory system” and “malignant neoplasm of bronchus and lung, unspecified,” respectively. No specific mention of mesothelioma was recorded on the death certificates.

In summary, no excess of malignant mesothelioma has been reported in any of the available epidemiological studies of talc miners and millers heavily exposed to talc. Although some of the individual cohorts were small, the collective number of workers followed was substantial (6) and the cohorts were followed for many decades. These workers were clearly and highly exposed to the talcs: all except the Norwegian cohort reported statistically significant excess mortality due to pneumonoconiosis, a group of non-malignant respiratory diseases caused by heavy exposure to dusts.

Ierardi and Marsh (58) conducted a power analysis to determine whether the pooled cohorts could detect a true association between talc exposure and mesothelioma risk among talc miners and millers, if there were one. Based on 130,154 total person-years of follow-up across five cohorts, the authors estimated that the pooled cohorts (30, 51, 53, 56) had 59% and 78% power to detect a 2.5-fold or greater and a 3.0-fold or greater increased risk of mesothelioma, respectively. Because the one malignant mesothelioma in the Vermont cohort was identified following a focused search of death certificates for the cohort but not the referent group, its inclusion results in a conservative bias. These findings were confirmed in Ierardi et al. (6), which reported an alternate pooled Standardized Mortality Ratio (SMR) of 0.242 (90% Confidence Interval [CI]: 0.012–1.15) with additional follow up time but no new deaths. When considered together, the moderate confidence in study findings, large study populations, long duration of follow up, and consistency of null findings indicate that talc exposure is not associated with mesothelioma.

#### Epidemiological findings for lung cancer

Among the most recent mortality updates for the talc miner and miller cohorts (1, 30, 43, 53, 56), Honda et al. (43) was the only study to report a statistically significant excess risk of lung cancer (SMR = 2.32; 95% CI: 1.57–3.29). Lung cancer mortality was slightly elevated, but did not achieve statistical significance, in the French (SMR = 1.23; 95% CI: 0.76–1.89) and Vermont (SMR = 1.44; 95% CI: 0.98–2.03) talc miners and millers but was close to unity for the Austrian (SMR = 1.06; 95% CI: 0.43–2.19), Italian (SMR = 1.02; 95% CI: 0.82–1.27) and Norwegian (SIR = 1.17; 95% CI: 0.73–1.79) talc miners and millers (1, 30, 53, 56).

Most of the available studies that evaluated lung cancers were rated medium quality. The higher quality studies generally did not demonstrate an association, or attributed observed increased risks to potential confounding by cigarette smoking, as risks did not correlate with indicators of talc exposure. Findings for each of the cohort studies that was rated high quality for exposure characterization are discussed more below.

Rubino et al. (50) followed 1,346 talc miners and 438 millers hired between 1921 and 1950 and that worked for at least 1 year in the Val Chisone talc operations. Mortality from lung cancer was slightly lower than expected for the entire cohort. Historical air sampling records were used to estimate cumulative dust exposure for each worker. Internal comparisons by exposure category showed that lung cancer mortality did not increase with increasing exposure for miners or millers. Coggiola et al. (48) updated the cohort and observed 44 lung cancers from 1946 through 1995 (SMR = 1.07; 95% CI: 0.73–1.50 for miners and SMR = 0.69; 95% CI: 0.34–1.23 for millers) (48). When stratified by duration of exposure, no exposure-response pattern was found (1, 48, 51).

Wild et al. (30) reported no increased lung cancer mortality among French (SMR = 1.23; 95% CI: 0.76–1.89) and Austrian (SMR = 1.06; 95% CI: 0.43–2.19) talc mining and processing employees (30). Using detailed employment histories and a job exposure matrix, cumulative talc exposures were estimated, and each cohort member was assigned a concentration of low (2.5 mg/m^3^), medium (10 mg/m^3^), or high (40 mg/m^3^). A nested case-control analysis showed no association of lung cancer with increasing cumulative exposure to talc, after adjusting for smoking, exposure to quartz, and underground work (30). The authors reported a reduced exposure odds ratio (OR = 0.73; CI not reported) for cohort members in the highest cumulative talc exposure group (≥800 mg/m^3^-years).

Honda et al. (43) reported 31 deaths from lung cancer among 809 New York talc miners and millers who had worked at least 1 day between 1948 and 1989 (SMR = 2.32; 95% CI: 1.57–3.29). The increased risk appeared to be limited to workers hired before 1955 (SMR pre-1955: 2.86; 95% CI: 1.90–4.14), as the SMR for workers hired after 1955 was 0.83 (95% CI: 0.17–2.42). The increased risk among those hired before 1955 was strongest among miners (SMR = 3.94; 95% CI: 2.33–6.22, based on 18 cases) and not elevated among millers (SMR = 1.28; 95% CI: 0.51–2.63, based on 7 cases). Lung cancer risk did not increase with increasing cumulative exposure categories after adjustment for age and years since hire (43). Relative to cohort members with the lowest cumulative respirable dust exposure (0 to <95.1 mg/m^3^), individuals in the two highest cumulative exposure groups had slightly reduced lung cancer risk (RR for cumulative exposure of 95.1-<987 mg/m^3^ = 0.8; 95% CI: 0.3–1.9, and RR for cumulative exposure of 987+ mg/m^3^ = 0.5; 95% CI: 0.2–1.3).

Exposure characterization was considered the most critical study quality domain in the quality evaluation of lung cancer studies. Three studies (30, 43, 50) were rated high quality for the exposure characterization domain. In the domain of confounding control, none of the studies had data on individual smoking histories. In order to increase cohort size, the New York cohorts did not limit eligibility based on minimum employment (e.g., 1 year) as is common in cohort mortality studies (42). This might have introduced confounding, as short-term or transient workers tend to have a higher prevalence of smoking and other health risk factors (46). Consequently, half of the talc workers in the mortality study conducted by Dement et al. (42) were employed <1 year (42). Stille and Tabershaw (47) updated the earlier cohort study and reported that all workers combined showed a moderate excess of lung cancer deaths (SMR = 1.57; no CI reported) (47). Smoking histories were not available for many workers, but all lung cancer cases were known to have been cigarette smokers (31).

The impact of prior occupational exposure to respiratory carcinogens also was not considered in most studies. One exception was Stille and Tabershaw (47), which presented stratified analyses for workers with and without known prior employment where exposure to occupational lung carcinogens might have occurred. When stratified by employment history, talc workers with known prior employment also showed excess lung cancer mortality (SMR = 2.14; no CI reported), and talc workers with no known work prior to their talc facility employment showed a slight deficit of lung cancer deaths (SMR = 0.76; no CI reported). An increased risk of death from lung cancer also was restricted to short-term workers in the updated cohort analysis (46). Among 705 white men employed between 1957 and 1977, lung cancer mortality was statistically significantly elevated among workers employed < 1 year (SMR = 3.17, based on six lung cancer deaths). The authors attributed the excess lung cancer to previous employment and smoking (46).

In summary, lung cancer mortality was not elevated among most of the cohorts of talc miners and millers exposed to high levels of respirable talcs and accessory minerals. While some cohort mortality studies reported an association between occupational talc exposure and lung cancer mortality, additional epidemiological investigations reported no such association or attributed observed increased risks to potential confounding by cigarette smoking, as risks did not correlate with indicators of talc exposure (43–47). Specifically, individual study quality ratings were high overall and the studies rated as high quality for the exposure characterization domain failed to demonstrate exposure-response patterns between occupational talc exposure and lung cancer mortality (30, 49). Given the moderate confidence in study quality ratings and the lack of a consistent association between occupational talc exposure and lung cancer mortality, the available epidemiological evidence does not demonstrate a causal association between talc exposure and lung cancer mortality.

### Evidence integration and hazard characterization

Our conclusions regarding hazard for each cancer type, based on the IOM classification system (8), are described below and in the protocol, and visualized in Table 2. Of three experimental animal studies of inhaled talc, only one mesothelioma was reported in a strain of rats prone to spontaneous mesothelioma. Genotoxicity studies were negative, and the few identified *in vivo* and *in vitro* mechanistic studies did not report strong evidence of the postulated chronic inflammation-mediated MOA in respiratory tissues at human-relevant exposure levels. The body of epidemiological literature evaluating talc exposure and risk of malignant mesothelioma, which consists of highly exposed workers with long follow up periods, fails to demonstrate any increased risk of malignant mesothelioma. Integrating the evidence demonstrating a lack of statistically significant increases in mesothelioma in inhalation rodent bioassays, the null findings in the higher-quality epidemiological literature, and the lack of evidence of a plausible MOA, we conclude that there is suggestive evidence of no association between inhaled talc and mesothelioma at human-relevant exposure levels.

**Table 2:** Evidence integration summary judgment: lung cancer and mesothelioma.

**Summary of animal, human, and mechanistic evidence**					**Inference across** **evidence streams**
**Evidence from studies of exposed humans**					*Suggestive Evidence of no association* • Several medium or high-quality epidemiological studies demonstrate no positive association between talc and LC or mesothelioma. • Three of four studies show no evidence of increased lung tumors • Single positive finding in one species exposed to doses associated with particle overload • Other inferences: • Talc is not DNA reactive • Insufficient evidence supporting a MOA
**Studies, outcome** **and confidence**	**Key findings**	**Factors that** **increase certainty**	**Factors that** **decrease certainty**	**Summary strength** **of evidence** **judgment**	
Lung Cancer and Mesothelioma fifteen *medium quality* prospective cohort studies: and one cohort study each in the high and low quality categories	• No elevated risk of LC largely consistent across studies • Few sporadic mesothelioma cases • Include both cosmetic and industrial talc	• Medium quality studies • Highly-exposed millers and miners in several countries • Semi-quantitative exposure characterization in some studies	• Smoking not adequately considered in LC studies • Previous employment not often considered in LC studies	Evidence against	
**Evidence from** ***in vivo*** **animal studies**					
**Studies, outcomes,** **and confidence**	**Key findings**	**Factors that** **increase certainty**	**Factors that** **decrease certainty**	**Summary strength** **of evidence** **judgment**	
Four *high-quality* studies in rats and mice	• Three studies with no lung tumors or mesothelioma • One study with > lung tumors in female rats	• High quality studies	• Particle overload/ exceedance of MTD in positive study • Micronized talc not relevant to human exposure	Indeterminate Micronized talc causes lung tumors in one species and sex of animals at doses >MTD conditions	
**Mechanistic Evidence or Supplemental Information**					
**Biological events** **or pathways (or** **other information** **category)**	**Primary evidence** **evaluated**	**Key findings,** **interpretation,** **and limitations**	**Evidence stream** **summary**		
Genotoxicity, chronic inflammation	• Three GLP/*guideline (K = 1)* genotoxicity studies • Two medium quality (*K* = 2) *in vivo* studies	• Rapid clearance of talc from lungs • Increased macrophages and markers of inflammation *in vitro* and *in vivo* • One study used intratracheal instillation, not relevant to humans	• Available mechanistic evidence is insufficient to support any mode (or modes) of action for talc and lung cancer		

In animal studies, a statistically significant increase in lung tumors was observed after inhalation of talc, although these tumors were limited to females of one species. Lung tumors occurred at very high administered doses that appeared to cause particle overload and using micronized talc (not typical of talc mining or milling exposures, or of cosmetic talcum powder products), limiting the ability to extrapolate these findings to relevant exposures in humans. Two animal studies examining inflammation in the lungs reported increased early inflammatory markers. Specifically, markers of inflammation and injury were observed after intratracheal instillation of large doses of talc, exposure conditions that do not correlate with those of humans. Other studies have reported increases in macrophages and other inflammatory markers after whole-body exposures, but again only with high talc concentrations.

The body of epidemiological evidence does not demonstrate a clear or consistent increase in mortality from lung cancer among talc miners and millers. A few studies reported excess lung cancer mortality, but lung cancer mortality risk did not increase with increasing talc exposure and the association potentially was confounded by smoking. In contrast to the lack of excess lung cancer deaths among talc miners and millers, a strong and consistent association has been observed between occupational talc exposure and non-malignant respiratory disease (NMRD), including pneumoconiosis (30, 43, 49, 56). A three-fold excess of mortality due to NMRD was reported in the earliest cohort study of New York talc workers, reflecting the high concentrations of dust present in the workplace; prior to 1948, median exposures ranged from 61 to 1,196 mppcf (45). Similarly, in the latest update of the Italian cohort, excess mortality for pneumoconiosis (SMR = 9.55; 7.43–12.1) was observed among talc miners and millers (1). These reported results for exposure and NMRD mortality underscore the magnitude of historical exposures and consequent health risks. Considering the totality of the evidence, we conclude that there is suggestive evidence of no association between inhaled talc and lung cancer at human-relevant exposure levels.

## Discussion

Our systematic review of talc and pulmonary cancers generated suggestive evidence of no association for exposure to talc and lung cancer and pleural mesothelioma. The body of epidemiological evidence is reasonably large and robust for lung cancer and mesothelioma and provides the most weight in the evidence integration, complemented by the number of high-quality experimental animal carcinogenicity bioassays, as well as the lack of convincing mechanistic evidence. Although the paucity of mechanistic information remains a limitation, the balance of evidence, especially the volume of epidemiological evidence demonstrating no increased cancer risks associated with even the highest “real world” human-relevant exposures, strengthens the current analysis.

The conclusions we reached in this systematic review are similar to those of IARC. Specifically, our findings for pulmonary cancers are consistent with IARC's classification of inhaled talc not containing asbestos or asbestiform fibers as “not classifiable” as to its carcinogenicity, in that neither identified any clear increase in cancer risk in animals and humans, and no clear MOA for carcinogenesis was identified (4). Although one meta-analysis (5) reported a statistically significant meta-SMR of 1.45 (95% CI: 1.22–1.72) for non-asbestiform talc and lung cancer, this review included several occupational cohorts with mixed exposures to possible lung carcinogens such as silica, asbestos, and radon (miners) resulting in high heterogeneity across reported study results. However, considering several newer studies, combined with a more rigorous systematic review methodology, our evaluation differed from these, indicating suggestive evidence of no association.

Further, the physicochemical properties of non-fibrous talc (e.g., inertness) and recent evaluations of potential MOAs indicate talc poses little to no concern for carcinogenic effects in humans, especially at less than lung overload exposures. It also is worth noting that despite claims that some talcs and therefore some talcum powders contain trace amounts of asbestiform minerals, high occupational exposures leading to large excesses of pneumoconiosis deaths were not associated with increased mortality from lung cancer, and these studies consistently report no excess (in fact, no cases) of malignant pleural mesothelioma.

One strength of our review is that we drew from the strongest aspects of established methodologies of several organizations' systematic review guidance in an attempt to provide a full and transparent evaluation. We recognize, however, that there still may be areas where further refinement in the approach is possible.

In sum, and based on the integration of evidence from animal experiments, mechanistic evaluations, and epidemiological studies all of reasonable methodological quality, it is unlikely that talc and cosmetic talcum powders at human-relevant exposures cause human pulmonary cancers, including lung cancer and mesothelioma.

## Data availability statement

The additional study results are presented in the article/Supplementary material; further inquiries can be directed to the corresponding author.

## Author contributions

Conception/scoping: KM and HL. Analysis of evidence: HL, DL, OL, RF, AI, WT, JC, AU, and KC. Wrote sections of manuscript: HL, KM, WT, RF, AU, AI, OL, KC, JC, and DL. QA/review: MC, PB, and KM. All authors contributed to manuscript revision, read, and approved the submitted version.

## Funding

This work was sponsored by the Center for Truth in Science, a 501(c)3 non-profit organization.

## Conflict of interest

Authors HL, KM, WT, DL, OL, JC, KC, RF, MC, AI, and AU are employed by Stantec ChemRisk, a consulting firm that provides scientific support to the government, corporations, law firms, and various scientific/professional organizations. Authors KM, WT, MC, and AI have been retained as expert witnesses on behalf of defendants in litigation matters in which it has been alleged that products containing talc caused mesothelioma or other cancers. Author PB is Full Professor at the Renaissance School of Medicine at Stony Brook University and Department of Medical and Surgical Sciences, University of Bologna, and Senior Scientific Advisor to ChemRisk; he has no conflicts to declare. The content and the conclusions of the manuscript are exclusively those of the authors.

## Publisher's note

All claims expressed in this article are solely those of the authors and do not necessarily represent those of their affiliated organizations, or those of the publisher, the editors and the reviewers. Any product that may be evaluated in this article, or claim that may be made by its manufacturer, is not guaranteed or endorsed by the publisher.
