# Supplementary material for: Systematic review of the scientific evidence of the pulmonary carcinogenicity of talc
Source: Front Public Health. 2022 Oct 11;10:989111. doi: 10.3389/fpubh.2022.989111 (PMC9593030; doi:10.3389/fpubh.2022.989111)
Supplement: Supplementary file 1 [file Table_1.DOCX]

# **Supplementary Table – Data Extraction Results**

| Full Citation | Type of Talc | Talc Content (%) | Other Mineral Content (%) | Other Notes on Talc Under Study | Animal Model | Route of Exposure | Exposure Concentration | Exposure Duration | Type of Assays Performed | Significant Response Above Control (Y/N) | No. of Mesotheliomas vs. Controls | No. of Lung Cancers vs. Controls | No. of Fibrotic Disease vs. Controls | Significant Effects |
| --- | --- | --- | --- | --- | --- | --- | --- | --- | --- | --- | --- | --- | --- | --- |
| Keskin, N., Y. A. Teksen, E. G. Ongun, Y. Ozay, and H. Saygili. 2009. Does long-term talc exposure have a carcinogenic effect on the female genital system of rats? An experimental pilot study. Arch Gyn Obstet 280 (6):925-931. | Only stated "talc" | Not listed | Not listed | Not listed | Rats | Intravaginal and perineal aerosol application | 100 mg in 0.5 mL saline | Daily for 3 months | Histological Assessment | N | 0 | 0 | 0 | There was no evidence of neoplastic or preneoplastic change in any of the experimental groups although there was evidence of foreign body reaction/infection. |
| Wagner, J. C., G. Berry, et al. (1979). "An animal model for inhalation exposure to talc." Dusts and Disease: Proceedings of the Conference on Occupational Exposures to Fibrous and Particulate Dust and Their Extension into the Environment. R. Lemen and J. M. Dement. Park Forest South, IL, Pathotox Publishers: 389-392 | Italian 00000 grade (Northern Italy) | 92% | 3% chlorite 1% carbonate 0.5-1% quartz | Non-fibrous talc No asbestos minerals of either the tremolite or chrysotile varieties have been detected in the many samples of this powder examined. Effects of this talc are compared with SFA chrysotile asbestos and non-treated controls. | Rat | Inhalation | 10.8 mg/m^3^ | 7.5 h/d, 5 d/wk for 3, 6, or 12 months | Lung dust burden, histological examination | Y | Talc: 0/81 Chrysotile: 1/80 Controls: 0/71 | Talc: 2/81 Chrysotile: 12/80 Control: 1/71 | See significant effects | Both Italian talc and SFA chrysotile produced fibrosis to a similar extent. There was evidence of progression after exposure had discontinued in animals with longer exposure. Talc exposure led to a mean fibrosis range of 2.2 (minimal) to 4.6 (slight), on a 7 point scale.  It was reported that “[f]or talc, the mean amounts of dust in the lungs were 2.8, 4.5 and 12.3 mg per rat at the end of exposures of 3, 6 and 12 months respectively.”  Two rats exposed to talc had adenomata of the lung. |
| Wehner, A. P., G. M. Zwicker, et al. (1977). "Inhalation of talc baby powder by hamsters." Food and Cosmetics Toxicology 15(2): 121-129. | High grade cosmetic talc powder  (from Vermont talc) | >95% | Trace carbonates, platy chlorite and rutile | The talc aerosol was generated from Johnson's Baby Powder, lot 228p, provided by Johnson & Johnson. Non-fibrous talc  Total talc aerosol concentration was measured daily, and compared to control rats in an identical chamber exposed to filtered room air. | Hamster | Inhalation | 8 μg/litre | 3, 30 or 150 min/day, 5 days/week for 30 days, or for 30 or 150 min/day until they died naturally (max 300 days) | Histological examination, X-ray fluorescence and  X-ray diffraction | N | n/a | Talc: 1/500 Control: 1/100 (See significant effects) | n/a | No significant differences observed when comparing the survival times of the exposed groups to each other and to controls. . However, it was noted that “the mean survival time of the males was significantly (P < 0.05) longer than that of the females” In all groups.  Based on the type, incident and severity of observed lesions, there was no indication of a dose-response relationship and no significant differences between the exposed groups and the controls. Only a few neoplasms were found, and their incidence was not related to treatment.   X-ray fluorescence and by X-ray diffraction showed deposition of talc particles in the lungs of exposed animals. |
| NTP (1993). "NTP Toxicology and Carcinogenesis Studies of Talc (CAS No. 14807-96-6) in F344/N Rats and B6C3F1 Mice (Inhalation studies)." Natl Toxicol Program Tech Rep Ser 421: 1-286 | 7023P-9108, Lot B 1842 | Not listed | Not listed | Non-asbestiform, cosmetic grade Manufactured by Pfizer, Inc. and is one of their microtalc series of products, top particle size of 10 μm | Rat | Inhalation | 0, 6, or 18 mg/m^3^ | 6 hrs/day, 5 days/week, up to 113 weeks (males) or 122 weeks (females) | Lung dust burden Histological Examination | Y | n/a | See significant effects | Male: 50/149 Female: 69/148 (interstitial fibrosis) | It was reported that “[i]nhalation exposure of rats to talc produced a spectrum of inflammatory, reparative, and proliferative processes in the lungs…Lung burdens were generally proportional to exposure concentration at each interim evaluation.”  There was increased incidence of benign or malignant pheochromocytomas of the adrenal gland in male rats.  There was increased incidences of alveolar/bronchiolar adenomas and carcinomas of the lung and benign or malignant pheochromocytomas of the adrenal gland in female rats. |
|  | MP 10-52 Grade talc | Not listed | Not listed |  | Mouse | Inhalation | 0, 6, or 18 mg/m^3^ | 6 hrs/day, 5 days/week, up to 104 weeks | Lung dust burden Histological Examination | N | n/a | None | n/a | It was reported that “[i]nhalation exposure [of mice] to talc was associated with chronic active inflammation and accumulation of macrophages in the lung.”  Lung burdens of mice exposed to 18mg/m^3^ were disproportionately greater than those of mice exposed to 6 mg/m^3^, suggesting impaired talc clearance.  There was no evidence of carcinogenic activity of talc in male or female mice exposed to 6 or 18 mg/m^3^ of talc. |
| Pickrell, J.A., et al. (1989). "Talc deposition and effects after 20 days of repeated inhalation exposure of rats and mice to talc." Environmental Research 49: 233-245 | Asbestos-free talc | Not listed | 19.3% Mg | 3 concentrations of asbestos-free talc, with filtered air control. Talc obtained from Midwest Research Institute (Kansas City, MO).  Exposed both rats and mice and measured talc accumulated in the lung. A simulation model and data for accumulated talc lung burdens were used to project lung burdens that would result from longer exposures. | Rat | Inhalation | 0, 2, 6, or 18 mg/m^3^ | 6hr/day, 5 days/week, for 4 weeks (20 days), starting at 6-7 weeks old | Lung dust burden Histological Examination Simulation Model | N | n/a | n/a | n/a | It was reported that “[t]he normalized lung burdens of both mice and rats were lower at the lowest exposure level than at the two higher exposure levels; however, the difference was significant only for the rats.”   No exposure-related lesions were observed in lung tissue “except for a modest, diffuse increase in free macrophages within alveolar spaces of both rats and mice exposed to the highest concentration of talc”  in rats, there were less macrophages were fewer and they were more diffusely scattered throughout the lung In mice, intraalveolar macrophages were focally aggregated.  Also, “[n]ormalized mean mouse lung burdens were 30 to 65% higher than rat lung burdens…Lung burdens of talc per gram lung were higher at all levels of talc exposure in mice than in rats, suggesting either a greater deposition or slower clearance of talc in the mice.” |
|  | Asbestos-free talc | Not listed | 19.3% Mg |  | Mouse | Inhalation | 0, 2, 6, or 18 mg/m^3^ | 6hr/day, 5 days/week, for 4 weeks (20 days), starting at 6-7 weeks old | Lung dust burden Histological Examination Simulation Model | N | n/a | n/a | n/a |  |

*Tremolite content was not reported in any of the studies listed in this table.
