# Supplementary material for: Systematic review of the scientific evidence of the pulmonary carcinogenicity of talc
Source: Front Public Health. 2022 Oct 11;10:989111. doi: 10.3389/fpubh.2022.989111 (PMC9593030; doi:10.3389/fpubh.2022.989111)
Supplement: Supplementary file 2 [file Table_2.DOCX]

**Supplementary Materials**

## Table of Contents

[Keskin (2009) 2](#_Toc83115550)

[NTP (1993) 3](#_Toc83115551)

[Pickrell (1989) 4](#_Toc83115552)

[Wagner (1977/1979) 5](#_Toc83115553)

[Wehner (1977) 6](#_Toc83115554)

# Keskin (2009)

| **Duration** | **Metric** | **Rating** | **Comments** |
| --- | --- | --- | --- |
| Test Substance | 1. Identity | 3 | Identified talc but no other details provided |
|  | 2. Source | 3 | Not reported |
|  | 3. Purity | 3 | Not reported |
| Test Design | 4. Negative and Vehicle Controls | 1 | 2 control groups, 1 with no intervention and 1 with saline |
|  | 5. Positive Controls | NR |  |
|  | 6. Assay Randomized Allocation | 3 | Not reported |
| Exposure Characterization | 7. Preparation and Storage of Test Substance | 4 | Not reported |
|  | 8. Consistency of Administration | 3 | Aerosolized talc applied across exposure groups but specifics not reported to allow for assessment of consistency |
|  | 9. Reporting of Doses/Concentrations | 2 | 100 mg in 0.5 mL saline but not mean or SD reported |
|  | 10. Exposure Frequency and Duration | 1 | Daily for 3 months |
|  | 11. Number of Exposure Groups and Dose Spacing | 2 | 4 exposure groups with different exposure types although rationale for spacing was not reported |
|  | 12. Exposure Route and Method | 1 | Aerosolized perineal and vaginal application |
| Test Organism | 13. Test Animal Characteristics | 3 | Animal characteristics were provided but source was not |
|  | 14. Adequacy and Consistency of Animal Husbandry Conditions | 2 | Light/dark cycle, food and water, and temperature provided |
|  | 15. Number per Group | 2 | 7 females per group |
| Outcome Assessment | 16. Outcome Assessment Methodology | 1 | Detailed histological examination |
|  | 17. Consistency of Outcome Assessment | 1 | Consistent for all animals |
|  | 18. Sampling Adequacy | 2 | Sampling number relatively low at 7 animals/group |
|  | 19. Blinding of Assessors | 2 | Not reported but unlikely to have an impact on outcome |
|  | 20. Negative and Control Response | 1 | Minimal level of inflammation relative to experimental groups |
| Confounding/Variable Control | 21. Confounding Variables in Test Design and Procedures | 1 | No difference in initial weight reported |
|  | 22. Health Outcome Unrelated to Exposure | 2 | No report of unrelated outcomes and impact on attrition although did not seem relevant |
| Data Presentation and Analysis | 23. Statistical Methods | 1 | Analysis using Fisher’s exact test |
|  | 24. Reporting of Data | 1 | Findings reported in detail for each group |

# NTP (1993)

| **Duration** | **Metric** | **Rating** | **Comments** |
| --- | --- | --- | --- |
| Test Substance | 1. Identity | 1 | Identified |
|  | 2. Source | 1 | Pfizer microtalc, obtained from Walsh Associates (lot (W101882 and B5415), Barretts MT mine |
|  | 3. Purity | 1 | “high purity”, characterized by two analytical chemistry labs, no asbestos detected |
| Test Design | 4. Negative and Vehicle Controls | 1 | One of the three exposure groups was 0 mg/m^3^ talc |
|  | 5. Positive Controls | NR |  |
|  | 6. Assay Randomized Allocation | 1 | Randomized into groups |
| Exposure Characterization | 7. Preparation and Storage of Test Substance | 1 | Stored in sealed bags at room temperature, detailed description of aerosol preparation |
|  | 8. Consistency of Administration | 2 | Consistent aerosol generator used, aerosol concentration monitored daily; authors reported that “while the overall means were very close to target concentrations, there were problems experienced in maintaining control of chamber concentrations” |
|  | 9. Reporting of Doses/Concentrations | 1 | Concentration mean reported and detailed in appendices |
|  | 10. Exposure Frequency and Duration | 1 | Rats: 6 hrs/day, 5 days/week, up to 113 weeks (males) or 122 weeks (females)  Mice: 6 hrs/day, 5 days/week, up to 103 or 104 weeks |
|  | 11. Number of Exposure Groups and Dose Spacing | 1 | 3 exposure groups: 0, 6, and 18 mg/m3 |
|  | 12. Exposure Route and Method | 1 | Full body inhalation |
| Test Organism | 13. Test Animal Characteristics | 1 | F344/N rats and B6C3F1 mice |
|  | 14. Adequacy and Consistency of Animal Husbandry Conditions | 1 | Rats and mice housed individually, Standard food and water |
|  | 15. Number per Group | 1 | 50 males and 50 females (rats and mice each) |
| Outcome Assessment | 16. Outcome Assessment Methodology | 1 | Clinical observations/body weights were measured weekly for 13 weeks, then monthly. Detailed autopsy performed on each animal at death; quality assessment protocol for pathology |
|  | 17. Consistency of Outcome Assessment | 1 | Consistent for all animals |
|  | 18. Sampling Adequacy | 1 | Every animal assessed equally with sufficient n for conclusions |
|  | 19. Blinding of Assessors | 2 | Not reported if done |
|  | 20. Negative and Control Response | 1 | Control results reported |
| Confounding/Variable Control | 21. Confounding Variables in Test Design and Procedures | 1 | Consistency across experimental groups at start |
|  | 22. Health Outcome Unrelated to Exposure | 1 | None reported |
| Data Presentation and Analysis | 23. Statistical Methods | 1 | Statistical testing performed, and fully defined |
|  | 24. Reporting of Data | 1 | Findings reported in detail for each group, extensive appendices |

# Pickrell (1989)

| **Duration** | **Metric** | **Rating** | **Comments** |
| --- | --- | --- | --- |
| Test Substance | 1. Identity | 1 | Identified |
|  | 2. Source | 2 | Obtained from Midwest Research Institute (Kansas City, MO), a subcontractor  of the National Toxicology Program, NIEHS but no additional details given |
|  | 3. Purity | 2 | The talc contained 19.2 to 19.4% magnesium (Mg) and was free of asbestos |
| Test Design | 4. Negative and Vehicle Controls | 1 | Control groups of equal conditions except exposure dose were included for rats and mice |
|  | 5. Positive Controls | NR |  |
|  | 6. Assay Randomized Allocation | 1 | Randomized by weight |
| Exposure Characterization | 7. Preparation and Storage of Test Substance | 2 | Description of preparation of aerosol generator, no description of talc storage |
|  | 8. Consistency of Administration | 1 | Consistent aerosol generator used; aerosol concentration measured continuously through exposure and particle size measured to monitor accuracy |
|  | 9. Reporting of Doses/Concentrations | 1 | Concentration mean and SD reported as well as particle size mean and SD |
|  | 10. Exposure Frequency and Duration | 1 | Each exposure group was exposed 6 hr/day, 5 days/week for a total of 20 exposure days |
|  | 11. Number of Exposure Groups and Dose Spacing | 2 | 3 exposure groups and 1 control group each for mice and rats, rationale for dose and exposure spacing not specified |
|  | 12. Exposure Route and Method | 1 | Inhalation exposure using multitiered inhalation chambers with internal volumes of either 0.9 or 1.7 m3 |
| Test Organism | 13. Test Animal Characteristics | 2 | Reported age, sex and source but not on starting weight and health status |
|  | 14. Adequacy and Consistency of Animal Husbandry Conditions | 1 | Temperature, humidity, airflow rate, food and water rates were all reported although no report of light-dark cycle |
|  | 15. Number per Group | 1 | 10 mice or rats/sex/exposure |
| Outcome Assessment | 16. Outcome Assessment Methodology | 1 | Measured lung burden and detailed histological examination |
|  | 17. Consistency of Outcome Assessment | 1 | Consistent for all animals |
|  | 18. Sampling Adequacy | 2 | 5 male and 5 female mice and rats were sacrificed from each exposure group for lung burden and histological analysis, somewhat small n |
|  | 19. Blinding of Assessors | 2 | Not reported but unlikely to impacts results |
|  | 20. Negative and Control Response | 1 | Results for lung burden reported for control group; no exposure related abnormalities observed at necropsy at high doses so low dose exams not done |
| Confounding/Variable Control | 21. Confounding Variables in Test Design and Procedures | 2 | Lung weight and exposure concentration were controlled for, rats and mice were either 6 or 7 weeks old at start of experiment – age not controlled for |
|  | 22. Health Outcome Unrelated to Exposure | 2 | Variance in lung burden between rats and mice but authors state that it is possible this is due to increased deposition in one species |
| Data Presentation and Analysis | 23. Statistical Methods | 1 | Statistical analysis methods reported |
|  | 24. Reporting of Data | 2 | Results by sex not reported but may not impact results |

# Wagner (1977/1979)

| **Duration** | **Metric** | **Rating** | **Comments** |
| --- | --- | --- | --- |
| Test Substance | 1. Identity | 1 | Identified |
|  | 2. Source | 1 | Italian 00000 grade (Northern Italy) |
|  | 3. Purity | 1 | 92% talc; no asbestos found in previous analyses of this grade of talc |
| Test Design | 4. Negative and Vehicle Controls | 2 | Negative control group included; not clear but seems as if just an untreated group |
|  | 5. Positive Controls | 1 | Superfine Chrysotile asbestos treatment group |
|  | 6. Assay Randomized Allocation | 1 | Randomized into groups |
| Exposure Characterization | 7. Preparation and Storage of Test Substance | 2 | No description of storage, detailed description of aerosol preparation |
|  | 8. Consistency of Administration | 1 | Aerosol generated was measured daily using a gravimetric dust sampler, and variations were allowed for by adjusting the concentrations on the following days, so that the required dosage, calculated as the product of concentration and time, was achieved uniformly in a specific time. |
|  | 9. Reporting of Doses/Concentrations | 1 | 10.8 mg/m^3^ |
|  | 10. Exposure Frequency and Duration | 1 | 7.5 hours/day, 5 days/week for 3, 6, or 12 months |
|  | 11. Number of Exposure Groups and Dose Spacing | 2 | 3 exposure groups: talc, chrysotile, and untreated. Talc and chrysotile groups exposed to same total dust concentration; justification for spacing not provided |
|  | 12. Exposure Route and Method | 1 | Full body inhalation |
| Test Organism | 13. Test Animal Characteristics | 2 | Wistar rats; Starting weight reported but other characteristics were not, not likely to impact results |
|  | 14. Adequacy and Consistency of Animal Husbandry Conditions | 1 | Animals housed in groups of 4, treated in groups of 6, Standard food and water, filtered air for animal house overall |
|  | 15. Number per Group | 1 | 48 rats in each group: talc, chrysotile, untreated. In each treatment group, 24 animals for 3 months, 12 animals for 6 months, 12 animals for 12 months |
| Outcome Assessment | 16. Outcome Assessment Methodology | 1 | Full necropsy assessment performed. Lung fibrosis scored |
|  | 17. Consistency of Outcome Assessment | 1 | Consistent for all animals |
|  | 18. Sampling Adequacy | 1 | Every animal assessed equally with sufficient n for conclusions |
|  | 19. Blinding of Assessors | 1 | Lungs were examined in random order, without knowledge of the dust or length of exposure |
|  | 20. Negative and Control Response | 1 | Control results reported for both control groups |
| Confounding/Variable Control | 21. Confounding Variables in Test Design and Procedures | 2 | The “majority” of the rats were between 6 and 8 weeks old; no specific details provided |
|  | 22. Health Outcome Unrelated to Exposure | 1 | 1 rat had widespread lumphosarcoma which is independent of treatment |
| Data Presentation and Analysis | 23. Statistical Methods | 3 | No Statistical Testing |
|  | 24. Reporting of Data | 2 | Limited outcomes assessed: lung fibrosis score and lung tumor incidence |

# Wehner (1977)

| **Duration** | **Metric** | **Rating** | **Comments** |
| --- | --- | --- | --- |
| Test Substance | 1. Identity | 1 | Identified |
|  | 2. Source | 1 | Johnson’s Baby Powder, lot 228p, sourced from Vermont |
|  | 3. Purity | 1 | “over 95% w/w platey talc with trace quantities of carbonates (magnesite and dolomite) as well as platey chlorite and rutile” |
| Test Design | 4. Negative and Vehicle Controls | 1 | One “sham” exposure group for each exposure duration period |
|  | 5. Positive Controls | NR |  |
|  | 6. Assay Randomized Allocation | 1 | Randomly dividing animals into groups |
| Exposure Characterization | 7. Preparation and Storage of Test Substance | 2 | Description of preparation of aerosol generator, no description of talc storage |
|  | 8. Consistency of Administration | 1 | Consistent aerosol generator used, aerosol concentration measured daily, weight and chemical analysis to monitor accuracy |
|  | 9. Reporting of Doses/Concentrations | 1 | Concentration mean and SD reported |
|  | 10. Exposure Frequency and Duration | 1 | Two durations (30 days, 300 days), three frequencies (3 min/day, 30 min/day, 150 min/day). Note: No 3 min/day group for 30 day duration |
|  | 11. Number of Exposure Groups and Dose Spacing | 1 | 5 exposure groups and 2 control groups (1 for each study duration); study design based on past infant-exposure simulations |
|  | 12. Exposure Route and Method | 1 | Inhalation; single-tier exposure chamber for four cages |
| Test Organism | 13. Test Animal Characteristics | 1 | E1a:ENG strain Syrian Golden Hamsters |
|  | 14. Adequacy and Consistency of Animal Husbandry Conditions | 1 | Separate and identical (temp + humidity) rooms for control vs experimental; standard food and water |
|  | 15. Number per Group | 1 | 50 males and 50 females in experimental groups, 25 males and 25 females in control groups |
| Outcome Assessment | 16. Outcome Assessment Methodology | 1 | Body weights were measured every 2-3 wk during the growing period and every 4 wk thereafter. Detailed autopsy performed on each animal |
|  | 17. Consistency of Outcome Assessment | 1 | Consistent for all animals |
|  | 18. Sampling Adequacy | 1 | Every animal assessed equally with sufficient n for conclusions |
|  | 19. Blinding of Assessors | 3 | Not reported if done |
|  | 20. Negative and Control Response | 1 | Control results reported |
| Confounding/Variable Control | 21. Confounding Variables in Test Design and Procedures | 2 | Slight differences in weight at beginning of study were not adjusted for; 30-day hamsters started at age 4 weeks and 300-day started at age 7 weeks |
|  | 22. Health Outcome Unrelated to Exposure | 2 | Valvular endocarditis observed in some animals, attributed to unrelated infection |
| Data Presentation and Analysis | 23. Statistical Methods | 2 | Statistical testing performed, but the type of tests was not always reported |
|  | 24. Reporting of Data | 1 | Findings reported in detail for each group |
