# Supplementary material for: Systematic review of the scientific evidence of the pulmonary carcinogenicity of talc
Source: Front Public Health. 2022 Oct 11;10:989111. doi: 10.3389/fpubh.2022.989111 (PMC9593030; doi:10.3389/fpubh.2022.989111)
Supplement: Supplementary file 7 [file Data_Sheet_1.docx]

**Protocol for the systematic review of the scientific evidence on the carcinogenicity of talc**

Heather Lynch^1*^; Dan Lauer, MPH^2^; Olivia Leleck, MPH^3^; Rachel D. Freid, MPH^1^; Collins, J. MS^4^; Chen, K., MPH^4^; William Thompson, MS^1^; A. Michael Ierardi, MES, MS^4^; Ania Urban, PhD, MPH^5^; Michael Anthony (Tony) Cappello, PhD^2^; Paolo Boffetta, MD^6,7^; Kenneth A. Mundt, PhD, FACE^1^

*Corresponding Author: Heather Lynch, [heather.lynch@cardno.com](mailto:heather.lynch@cardno.com)

*^1^ Cardno ChemRisk; Boston, MA*

*^2^ Cardno ChemRisk, Boulder, CO*

*^3^ Cardno ChemRisk, Pittsburgh, PA*

*^4^ Cardno ChemRisk, Brooklyn, NY*

*^5^ Cardno ChemRisk, San Francisco, CA*

*^6^ Stony Brook Cancer Center, Stony Brook, NY*

*^7^ Department of Medical and Surgical Sciences, University of Bologna, Bologna, Italy*

Support: This work was funded by a grant from the Center for Truth in Science (CtiS).

Table of Contents

[**1.** **Introduction** 2](#_Toc69210651)

[**1.1.** **Rationale** 2](#_Toc69210652)

[**1.2.** **Objective** 2](#_Toc69210653)

[**2.** **Methods** 2](#_Toc69210654)

[**2.1.** **Eligibility Criteria** 3](#_Toc69210655)

[**2.2.** **Information Sources and Search Strategy** 4](#_Toc69210656)

[**2.3.** **Study Records: Data Abstraction** 5](#_Toc69210657)

[**2.4** **Study Quality Evaluation** 5](#_Toc69210658)

[**2.4.** **Evidence Synthesis** 7](#_Toc69210659)

[Epidemiology 7](#_Toc69210660)

[Experimental Animal and Mechanistic Information 8](#_Toc69210661)

[**2.5.** **Evidence Integration** 8](#_Toc69210662)

[**2.6.** **Risk Characterization, Uncertainties, and Implications** 9](#_Toc69210663)

1. **Introduction**
   1. **Rationale**

Talc (hydrated magnesium silicate) is neither an amphibole mineral nor a form of asbestos, but the mined mineral talc will contain various amounts and forms of accessory minerals. Pharmaceutical-grade talc is the most pure form of talc (minimum of 99% talc) followed by cosmetic-grade talc and industrial-grade talc. Industrial talc is reported to contain a variety of accessory minerals including tremolite, anthophyllite, carbonate and a small amount of crystalline silica. It has also been reported that some cosmetic talc as well as finished talcum powders may have been contaminated with asbestiform minerals; however, much controversy surrounds the methods used to correctly identify and quantify asbestos fibers (Fiume et al. 2015).^^[[1]](#footnote-1)^^ The published literature generally fails to demonstrate that talc and consumer products containing talc (such as body powders) increase the risk of any human cancer. Select studies, however, have been used by policymakers, the public, and attorneys, among others, to link talc with human health effects, including cancer. This highlights the importance and need for an objective and transparent systematic review of the scientific literature with a primary focus on the epidemiological evidence – but also addressing and integrating evidence from key animal and mechanistic studies – that plainly sets forth the most valid interpretations and conclusions and the scientific basis for them.

- 1. **Objective**

The primary objective of this systematic review (SR) is to critically evaluate the possible relationship(s) between exposure to talc occupationally and from the use of talc-containing products (primarily talcum powders and cosmetics) and cancer(s). For each possible association, we will present the evidence supporting and detracting from causality. The focus of our systematic review will be on respiratory cancers (i.e., lung cancer and pleural mesothelioma) and female reproductive tract cancers (primarily ovarian cancers), but additional specific endpoints may be addressed should the literature review indicate that other endpoints warrant consideration.

1. **Methods**

This SR will be conducted in accordance with best practices and guidance – incorporating aspects from a number of recognized systems including the Institute of Medicine (IOM) framework^[[2]](#footnote-2)^, EPA’s *Application of Systematic Review in TSCA Risk Evaluations^^[[3]](#footnote-3)^^,* the National Toxicology Program’s Office of Health and Translation (OHAT) systematic review framework^^[[4]](#footnote-4)^^, guidance from the National Academies of Sciences, Engineering (NASEM), EPA, and others. The review also will adhere to and we will complete the PRISMA checklist for systematic reviews. While each of these frameworks is fit-for-purpose, the principles of systematic review essentially are the same across all of these. The general steps for our review are outlined in Figure 1 (below).

We will conduct a robust and transparent critical review with a strong focus on the quality assessment of each primary study, synthesizing and integrating evidence as possible across epidemiological, toxicological and disease mechanistic lines of inquiry. Specific strengths and limitations of the systematically identified body of literature as well as critical evidentiary gaps will be presented and discussed. Where any cancer hazards of talc exposure are identified, exposure levels and scenarios at which risk is increased will be described, and if appropriate, characterized by dose-response analyses. We also will outline briefly the regulatory and decision-making implications of all findings. All aspects of the systematic review will be documented in a final report, which will be structured as a manuscript to be submitted for publication. All drafts and final versions (as was done with this protocol) will be subject to the review and approval of our team experts, Drs. Calabrese and Boffetta.


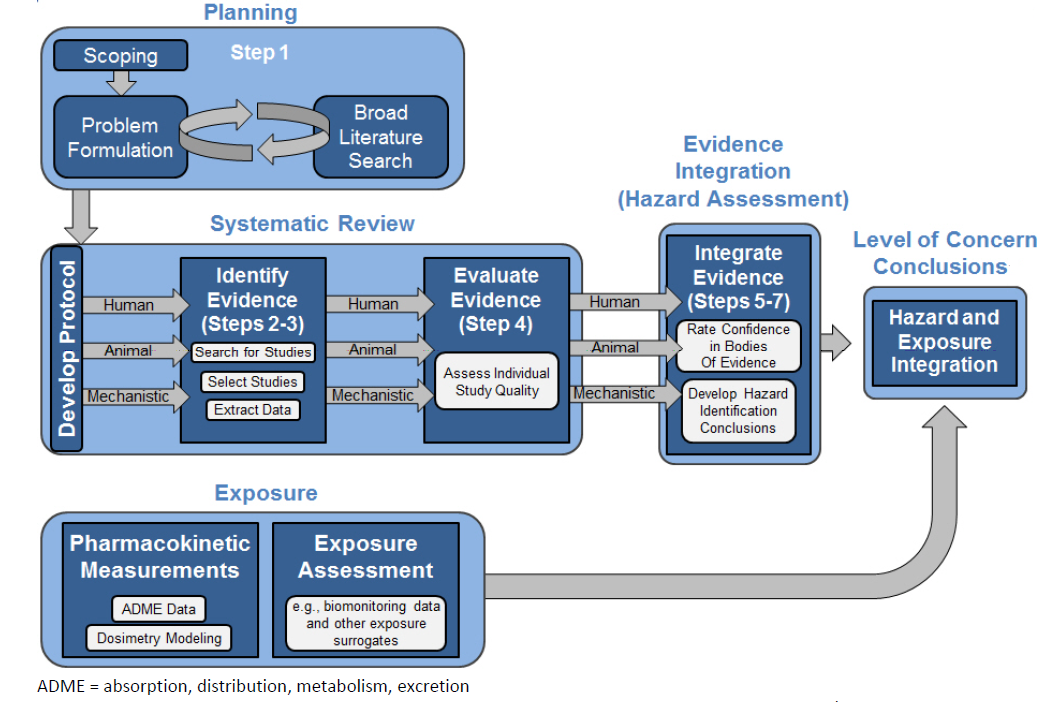


**Figure 1. OHAT Schematic for General Process**

- 1. **Article Eligibility Criteria**

Develop *a priori* a set of literature inclusion and exclusion criteria to identify the most relevant articles for full review consistent with systematic review principles.

**Table 1 PECO / Literature Screening Criteria**

| **Evidence Stream** | **Inclusion Criteria** | **Exclusion Criteria** |
| --- | --- | --- |
| Human | - Population: Humans, any study design, including case reports and case series - Exposure: talc specifically and/or asbestos in talc at any level/concentration; inhalation exposure or via migration through the external genital tract; chronic duration - Outcome: Any cancer, including mesothelioma, lung cancer, uterine, or ovarian cancer - Comparator: Unexposed or less-exposed human populations | - Oral and dermal studies - Substances other than talc or asbestos only studies - Mixture studies (e.g., co-exposures to talc and asbestos mixtures in industrial applications) - Talc studies related to mesothelioma treatments (e.g. pleurodesis) - Exposure not clearly specified - Non-English articles - Registry-based studies - Case reports/case series |
| Animal | - Population: Any mammalian species/strain - Exposure: All experimental animal studies with any level of controlled administration of talc via the inhalation, intravaginal exposure routes; chronic duration - Comparator: Unexposed animals - Outcomes: Any carcinogenic or chronic effects (e.g., pre-neoplastic changes) | - Ecotoxicity studies - Non-mammalian species (e.g., chicken, zebrafish) - Chemical mixture studies - Oral, dermal, intravenous or intraperitoneal exposure studies - Substances other than talc or asbestos only studies - Non-English articles |
| ADME and Mechanistic | - Population: any human and bacterial cells (*In vivo* and *in vitro* assays); all toxicokinetic studies - Exposure: All dose levels, durations and routes (as relevant to MOA) - Comparator: Negative/positive control groups, as needed - Outcome: Genotoxicity, key molecular initiating events or other steps in postulated MOAs (as identified in the problem formulation phase), particle deposition/clearance (toxicokinetic studies) | - Chemical mixture studies - Non-mammalian species (e.g., chicken, zebrafish) - Substances other than talc or asbestos only studies - Non-English articles |

- 1. **Information Sources, Search Strategy, and Selection Process**

We will conduct literature searches in PubMed, Web of Science and other sources, with support from our professional librarian, using existing agency reviews as a basis for cross-reference of critical references. Results of PubMed will be imported Microsoft Excel for the purposes of organization, screening, selection, and mapping to specific reviewers. Two reviewers will evaluate titles and abstracts for relevance and adherence to PECO. A summary flow chart depicting the total number of references and those excluded according to the criteria in Table 1 above will be provided.

The preliminary search string is as follows: (talc OR "talcum powder") AND ("cancer" OR carcinogen OR mesothelioma)

Animal and *In Vitro* Evidence

1. Experimental animal studies will be selected based on their overall relevance to the chronic health effects (primarily cancers), adherence to the PECO noted in Table 1 above, and relevance to the epidemiological literature. We will rely on publically available reviews and analyses of animal toxicology and *in vitro* data, including but not limited to the most recent reviews of talc from IARC and EPA.

2. We will conduct additional literature searches to capture peer-reviewed literature published after the body of literature considered in the IARC and EPA reviews was published.

Epidemiological Evidence

Literature searches initially will include coverage of groups or populations exposed to talc, including talc miners and millers (the groups historically the most highly exposed) as well as users of talcum powders and other products containing talc. Historically, talc and asbestos have been used in some of the same industries or for some of the same purposes, e.g., stiffening rubber products (IARC 2010). Consequently, studies of workers in settings where talc and asbestos have been used intentionally and concurrently (e.g., manufacture of rubber goods) are not useful for differentiating the risks individually associated with asbestos or talc, and will not be reviewed.

For all domains, the results of the initial literature searches will be screened for relevance to hazard evaluation and possibly quantitative risk characterization, if appropriate, according to the PECO criteria in Table 1 above. Those not selected will be identified based on the primary reason(s) for non-inclusion (e.g., not an epidemiological study, inadequate documentation of exposure or outcome, etc.), and classified accordingly for review transparency.

- 1. **Study Records: Data Abstraction**

Once the relevant body of literature is identified for each of the primary lines of evidence, each selected study will be reviewed, summarized in narrative form, and key studies tabulated by one researcher and verified/QCed by a second researcher. Study results will be considered in the context of the quality assessment, as described below. The methodological strengths and limitations of each key study’s results will be weighted for inclusion in the synthesis and integration steps.

## **2.4 Study Quality Evaluation**

We will follow a modified version of the study quality framework used by US EPA for the 2016 amended Toxic Substance Control Act (TSCA) risk evaluations, as outlined in *Application of Systematic Review in TSCA Risk Evaluations* (US EPA, 2018). This framework involves reviewing and rating studies according to six quality domains (e.g., outcome assessment and exposure characterization), each of which include 2-7 individual metrics assessing specific study features (e.g., temporality of the exposure metric) (Table 1 and 2 below). The specific requirements for each quality score (high, medium, low) and for each criterion is tailored to specific study designs (e.g., cohort studies and case-control/cross-sectional studies). Each study will be reviewed and evaluated for quality by two researchers.

**Table 1. Epidemiological Study Quality and Relevance in TSCA**

| **TSCA Domain** | **Metrics Included** |
| --- | --- |
| Study participation | Evaluation of participant selection methods, attrition, adequacy of the comparison group, performance bias |
| Exposure Characterization | Evaluation of reporting and adequacy of exposure measurement methods, exposure levels |
| Outcome Assessment | Evaluation of effect assessment methodology and reporting, including diagnostic methods, interview training, data sources (e.g., registries), and blinding |
| Potential Confounding / Variability Control | Evaluation of methodology and reporting of efforts to reduce research-specific bias, including matching, adjustment in multivariate models (including co-exposures, as relevant), stratification, etc. |
| Analysis | Evaluation of study design in terms of relevance to research question, as well as consideration of statistical power, reproducibility, statistical/modeling approaches |
| Other / Consideration for Biomarker Selection and Measurement | Specific to studies with biomarkers; includes considerations of storage and stability of samples, methods, and relationship of biomarker to external exposure or apical effects (for exposure and outcome biomarkers, respectively) |

**Table 2. Experimental Animal Study Quality and Relevance in TSCA**

| **TSCA Domain** | **Metrics Included** |
| --- | --- |
| Test Substance | Evaluation of the identity, purity, and properties of the test substance |
| Test Design | Evaluation of whether the design enables the study to distinguish the effect of exposure from other factors; includes randomization and use of controls |
| Exposure Characterization | Evaluation of reporting and adequacy of exposure measurement methods, exposure levels |
| Test Organism/Test Model | Evaluation of the animal model selected, number of animals, animal housing conditions, etc. |
| Outcome Assessment | Evaluation of effect assessment methodology and reporting |
| Confounding / Variability Control | Evaluation of whether experimental or analytical methods were used to control factors unrelated to exposure that could affect outcome (e.g., infections in the animal colony) |
| Data Presentation and Analysis | Evaluation of the appropriateness of statistical methods and reporting of all outcomes |

This analysis will not include the full numerical scoring schema as presented in the TSCA guidance document, as it tends not to afford adequate weight to the most important domains (e.g., exposure and adequate consideration of other risk factors as confounders). From our experience, under the current framework, significant flaws in one of the key domains in an otherwise highly rated study will not eliminate that study from the higher-quality category. In addition to the criteria that are focused on study methodological quality, we will consider publication bias across the body of selected studies.

Separately, existing systematic reviews will be assessed using a modified version of the AMSTAR^^[[5]](#footnote-5)^^ checklist, incorporating a relative rating system to assign studies as high, medium or low confidence. The identified systematic reviews will be considered and addressed in the discussion section of the manuscript should they provide additional context or inform overall integration of the scientific literature on talc and human health.

We will provide detailed tables summarizing the key studies and our study quality assessments in appendices and/or supplemental materials to the publication.

*Protocol update:*

*After extensive pilot testing of the adapted TSCA study quality evaluation, the following study quality evaluation process was conducted:*

*Detailed Study Quality Methods:*

All metrics in all domains initially were evaluated qualitatively (low, medium, or high). For each study, one investigator assigned a study quality score for each of the domain-specific metrics. Composite domain scores for TSCA domains containing three or more sub-domains (Study Participation, Exposure Assessment, Analysis) were determined by identifying the most frequent score across sub-domains. Overall domain scores for Outcome Assessment and Potential Confounding/Variable Control (two sub-domains) were assigned based on the most frequent sub-domain score, the median score (if high and low for two sub-domains), or the lowest score when two scores were adjacent (medium/low or high/medium). A second investigator reviewed the initial study quality scores, and any discrepancies were discussed and adjudicated. When the two investigators were unable to arrive at a consensus, a third reviewer was brought in to mediate any disagreements to arrive at a final determination.

Overall quality scores for each study were determined separately for case-control and cohort studies using a tiered approach in which the exposure characterization domain was initially evaluated; depending on whether this domain was sufficiently reasonably fulfilled (either medium or high-quality rating), Tier 2 included a weighing of scores on the remaining TSCA domains (see Supplemental Figures 1-2). TSCA guidelines dictate that an occupational cohort study contain detailed employment information facilitating the construction of a JEM, include a range and distribution of exposure sufficient to develop an exposure-response relationship, and establish an appropriate temporality between exposure and outcome (EPA 2018)

Experimental animal toxicology and mechanistic studies

For animal toxicology and selected mechanistic studies, we followed the TSCA study quality evaluation framework and assigned relative numerical ranks to each of the outcomes (1, 2 and 3 corresponding to high, medium and low) for each metric, then averaged the metric scores to arrive at an overall relative score of high, medium, or low quality. Mechanistic studies were evaluated according to Klimisch scoring (Klimisch, Andreae, and Tillmann 1997), a higher-level framework commonly used in regulatory decision-making that focuses on reliability, relevance and general adequacy of studies. When assessing reliability, reviewers judge adherence to or comparability to Organization for Economic Co-Operation and Development (OECD) guideline studies and adherence to Good Laboratory Practices (GLP). Separately, relevance and adequacy are judged considering the usefulness of the study for the purposes of the risk assessment – e.g., whether the testing strategy is aligned with physicochemical properties of the substance and whether the test species is relevant to the target species of interest.

*Protocol update*: After pilot testing of the AMSTAR framework on two reviews, it was determined that very little value was added and this methodology was not carried forward for additional studies.

*Protocol Update:* Shortly after beginning the study quality evaluation process, EPA released a revised systematic review protocol meant to supersede the 2018 TSCA systematic review guidance document. The study quality assessment criteria are almost identical to the 2018 criteria; the major change was the removal of the metric weighting factors and focus on numeric scoring. Therefore, we did not alter our tiered, adapted system – in fact, our methodology follows the new EPA TSCA systematic protocol even more closely than it followed the 2018 guidance.

- 1. **Evidence Synthesis**

Provide a detailed narrative whereby the results of human, animal, and mechanistic evidence are separately synthesized to determine the strength of the evidence for each line of scientific inquiry: robust, moderate, slight, indeterminate, or compelling evidence of no effect. The judgments are based loosely on those presented by US EPA (2021), and are informed by study quality and the fulfillment of Bradford Hill viewpoints including consistency, strength (magnitude) of effect, biological gradient/dose-response, and coherence across the individual lines of evidence.

For each line of evidence, a key feature of the evaluation and narrative will be to consider the study quality evaluation in the evidence integration and hazard conclusions. The narrative will include explanations of the relative contribution of methodological quality to the observed associations (i.e., whether certain study design features may have biased results); alternative factors and/or alternative hypotheses for any observed associations; and most importantly, the relevance of this set of literature and its findings to populations of interest (e.g., workers exposed to talc and consumers of products containing talc).

The synthesis conclusions for each realm of evidence and the basis for these conclusions, as well as the basis for the causality conclusions from the integration across the realms of evidence, will be summarized in tabular format. Each line of evidence will be assigned a qualitative descriptor of overall strength of the evidence. Specific considerations for each line of evidence are provided below.

Epidemiology

In order to evaluate consistency and coherence across the studies, present studies in table, figure, or other visual form to compare results by 1) study design and by 2) study quality classification (low, medium, high). For each study, evaluate whether the results indicate that talc is objectively and accurately measured or estimated, and associated with the disease(s) of interest. Specific factors to be considered include the strength and consistency of associations; freedom from serious bias, and degree to which alternative explanations were explored in that particular study (i.e., chance, bias and confounding) and reasonably can (or cannot) be discounted. Studies with higher methodological quality will be given more weight in the synthesis of the evidence.

If appropriate and given available data are amenable, a meta-analysis may be conducted, including an evaluation of possible publication bias. In some circumstances even when a summary relative risk estimate is not of interest or meaningful, forest plots can be helpful in visually presenting study results and the patterns visible across studies.

Experimental Animal and Mechanistic Information

As described in the Search Strategy section, the analysis of the animal and mechanistic information will be fit-for-purpose and focused on studies identified in recent agency reviews as well as more recent publications, for the cancer sites (i.e., lung, mesothelium and female reproductive system) addressed in the epidemiology section. We will consider the strength and consistency of the *in vivo* experimental animal studies (including consistency across species) for each cancer type. With regard to studies published after agency reviews, we specifically will consider whether the findings generally agree or diverge from the studies included in the agency reviews. We also will describe the available evidence for any postulated mechanisms, and weigh the relative strength of each, considering the availability and quality of data each postulated molecular initiating and key event in the MOA, characterizing any data gaps apparent in the MOA.

- 1. **Evidence Integration**

The results of the syntheses for each line of inquiry will then be weighted and integrated to reach clear conclusions regarding the potential cancer hazards of talc and talc contaminated by asbestos. In other words, the totality of the evidence will be synthesized within each line of inquiry (e.g., across all epidemiology studies) and then integrated with animal study findings and mechanistic considerations to reach conclusions on human cancer hazards. Again, we will draw upon the systematic review frameworks named above, but also refer to other frameworks focused specifically on causal interference (e.g., Adami et al., 2011).

This phase of the analysis will incorporate relevant Bradford Hill viewpoints, with particular emphasis on the biological plausibility of observed effects (considering the available MOA evidence) and possible dose-response relationships observed toxicologically and epidemiologically. The evaluation also will consider alternative explanations for the reported findings, particularly if the evidence from one line of inquiry contradicts that from another, with the greatest weighting afforded high-quality human evidence. Where possible, we will identify possible reasons why evidence conflicts (e.g., a mechanism of toxicity that operates in animals but not in humans).

The totality of evidence will be characterized according to IOM classifications for causation (incorporating fit-for-purposes modification, as necessary) detailed in the table below.


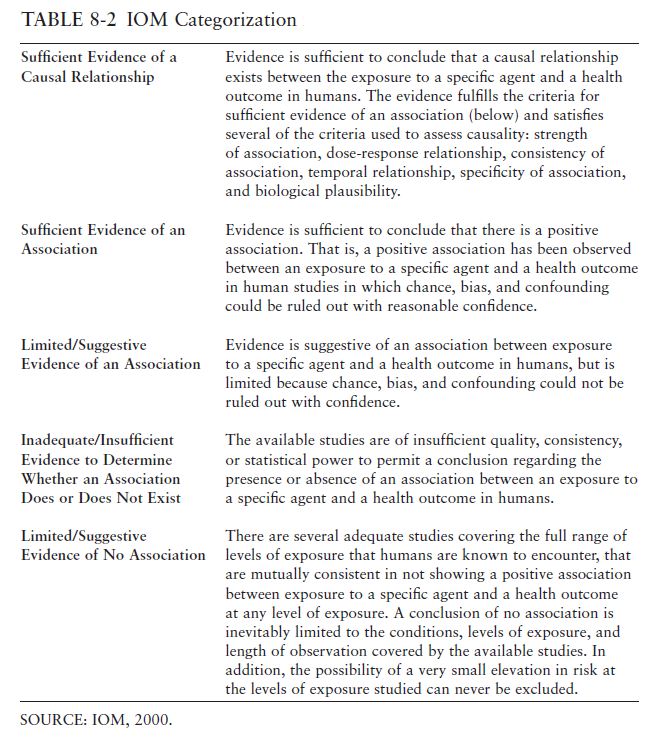


The results of the evidence integration and causal conclusions will be summarized in a tabular or other graphic format that highlights evidence for the primary basis for the judgment and any notable features, issues, or uncertainties, similar to the tabular summary proposed in the recent draft EPA Integrated Risk Information (IRIS) Handbook (EPA, 2020). A discussion of the level of certainty (or confidence) in the overall body of evidence for each cancer type and the resulting confidence in causal conclusions will also be provided.

- 1. **Risk Characterization, Uncertainties, and Implications**

Results of the evidence integration for the cancer hazards of talc, if any, further will be analyzed in the context of risk (i.e., dose-response relationship and levels at which cancer risks might be increased). Key gaps and uncertainties in the available scientific evidence, and how these might affect legal and political decision-making regarding talc, will be identified. Where possible, we will provide recommendations for further research and analysis to resolve remaining uncertainties. Lastly, we will discuss how the findings of our systematic review relate to previously published agency and peer-reviewed assessments of the human health effects of talc, carefully considering the relative strengths and weaknesses of those reviews in light of how faithfully their conclusions were based on sound systematic review methods including evidence synthesis and integration, and whether the conclusions should be revised based on more up-to-date published evidence. If appropriate, the results of the comparison of the reviews will be summarized in a tabular or other graphic format that highlight the different strengths and weaknesses.

The methods, data inputs and results of our systematic review of talc will be presented in a manuscript suitable for submission to a peer-reviewed journal. Prior to submission to the journal, the completed analysis will undergo internal review by all authors and internal editors and expert review Dr. Boffetta.

- 1. **Protocol Modifications**

During the review of tertiary literature (reviews and meta-analyses), the AMSTAR tool was applied to four studies. However, it became clear that the tool was not amenable to application to meta-analysis or critical reviews that were not conducted within a formal systematic review framework because, for example, AMSTAR criteria would necessitate rating all studies as “critically deficient.” No alternative standardized system for evaluating reviews and meta-analyses in depth (beyond a checklist, such as PRISMA) were identified. The decision was made to discuss any high-level strengths and weakness of the reviews, without a formal review process.

1. Fiume MM, Boyer I, et al. Safety Assessment of Talc as Used in Cosmetics. Int J Toxicol 2015; 34:66S-129S. [↑](#footnote-ref-1)
2. Institute of Medicine 2008. Improving the Presumptive Disability Decision-Making Process for Veterans. Washington, DC: The National Academies Press. https://doi.org/10.17226/11908. [↑](#footnote-ref-2)
3. United States Environmental Protection Agency (US EPA). 2018. Application of Systematic Review in TSCA Risk Evaluations. EPA Document # 740-P1-8001. Office of Chemical Safety and Pollution Prevention, US EPA, Washington, DC. [↑](#footnote-ref-3)
4. NTP. Handbook for Conducting a Literature-Based Health Assessment Using OHAT Approach for Systematic Review and Evidence Integration. March 4, 2019. Research Triangle Park, NC Office of Health Assessment and Translation (OHAT), Division of the National Toxicology Program, National Institute of Environmental Health Sciences; 2019. [↑](#footnote-ref-4)
5. Shea BJ, Grimshaw JM, et al. Development of AMSTAR: a measurement tool to assess the methodological quality of systematic reviews. BMC Med Res Methodol. 2007; 7:10.

   [https://amstar.ca/docs/Publication%20-%20Development%20of%20AMSTAR.pdf](https://urldefense.proofpoint.com/v2/url?u=https-3A__amstar.ca_docs_Publication-2520-2D-2520Development-2520of-2520AMSTAR.pdf&d=DwMFAg&c=QSj8pw-Dfe-PLjj4Ds2WCg&r=5oaanTdKzXuiG4Sw-9bRvZNIUuug3pCfily6C_qedzE&m=EYXZdpTr3LUMkb_uywARBLsJQUXhBi16Xn-uuZk7TcY&s=AKSRoexcnEZY0P6_4_ckzHm8R0DPluSwSI3r15O0ZLk&e=) [↑](#footnote-ref-5)
